# Supplementary material for: Causal Relationship between Aging and Anorexia Nervosa: A White-Matter-Microstructure-Mediated Mendelian Randomization Analysis
Source: Biomedicines. 2024 Aug 16;12(8):1874. doi: 10.3390/biomedicines12081874 (PMC11351342; doi:10.3390/biomedicines12081874)
Supplement: Supplementary file 1 [file biomedicines-12-01874-s001.zip › Supplementary Figure/Figure S8/Leaveoneout plot of MR analysis for TL to ubm-b-1495.pdf]

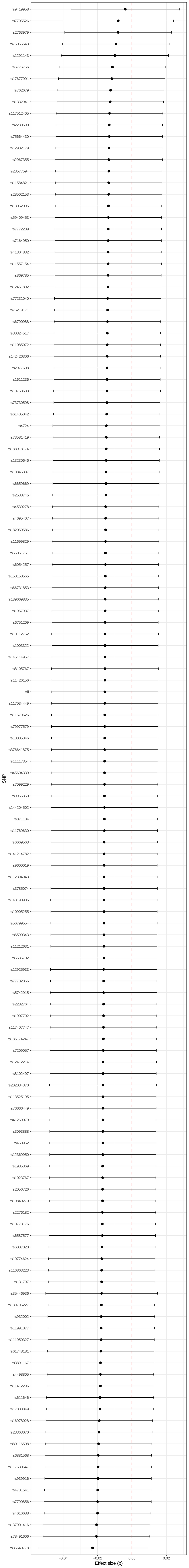
rs9419958
rs7705526
rs2763979
rs76065543
rs1291143
rs6776756
rs17677991
rs762679
rs1332941
rs117512405
rs2230590
rs75664430
rs12932179
rs2967355
rs28577594
rs11584821
rs28502153
rs13062095
rs59409453
rs7772289
rs7164950
rs41304832
rs11557154
rs869785
rs12451892
rs77231040
rs76219171
rs6790988
rs80324517
rs11085072
rs142426306
rs2977608
rs1611236
rs10768683
rs73730598
rs61405042
rs4724
rs73581419
rs188918174
rs13230646
rs10845387
rs6659669
rs2538745
rs4530278
rs4695407
rs182059586
rs11699829
rs56061761
rs6054257
rs150150565
rs66731853
rs139669835
rs1957937
rs6751209
rs10112752
rs1003322
rs145114957
rs8105767
rs11426156
A8
rs117034449
rs11579626
rs79977579
rs10805346
rs376641875
rs11117354
rs45604339
rs7099229
rs9955360
rs144204502
rs871134
rs11769630
rs6669563
rs141214782
rs9600019
rs112394943
rs3785074
rs143190905
rs10905255
rs56799554
rs6590343
rs11212631
rs6536702
rs12925933
rs77732866
rs5742915
rs2282764
rs1907702
rs117407747
rs185174247
rs7209057
rs12412214
rs8102497
rs202034370
rs113525195
rs76666449
rs41269079
rs3093888
rs450962
rs12369950
rs1985369
rs1023767
rs2056726
rs10840270
rs2276182
rs10773176
rs6587577
rs6007020
rs10774624
rs116863223
rs131797
rs35446936
rs139795227
rs932002
rs11991877
rs111050327
rs61748181
rs3891167
rs4498805
rs11412296
rs611646
rs17803849
rs16978028
rs28363070
rs80116508
rs6881568
rs117630647
rs939916
rs4731541
rs7790856
rs4616688
rs137901416
rs78491606
rs35640778
SNP
-0.04
-0.02
0.00
0.02
Effect size (b)
